# SUMOhydro: A Novel Method for the Prediction of Sumoylation Sites Based on Hydrophobic Properties

**DOI:** 10.1371/journal.pone.0039195

**Published:** 2012-06-14

**Authors:** Yong-Zi Chen, Zhen Chen, Yu-Ai Gong, Guoguang Ying

**Affiliations:** 1 Laboratory of Cancer Cell Biology, Tianjin Medical University Cancer Institute and Hospital, Tianjin, China; 2 Tianjin Key Laboratory of Cancer Prevention and Therapy, Tianjin Municipal Science and Technology Commission, Tianjin, China; 3 Bioinformatics Center, College of Biological Sciences, China Agricultural University, Beijing, China; Hospital for Sick Children, Canada

## Abstract

Sumoylation is one of the most essential mechanisms of reversible protein post-translational modifications and is a crucial biochemical process in the regulation of a variety of important biological functions. Sumoylation is also closely involved in various human diseases. The accurate computational identification of sumoylation sites in protein sequences aids in experimental design and mechanistic research in cellular biology. In this study, we introduced amino acid hydrophobicity as a parameter into a traditional binary encoding scheme and developed a novel sumoylation site prediction tool termed SUMOhydro. With the assistance of a support vector machine, the proposed method was trained and tested using a stringent non-redundant sumoylation dataset. In a leave-one-out cross-validation, the proposed method yielded an excellent performance with a correlation coefficient, specificity, sensitivity and accuracy equal to 0.690, 98.6%, 71.1% and 97.5%, respectively. In addition, SUMOhydro has been benchmarked against previously described predictors based on an independent dataset, thereby suggesting that the introduction of hydrophobicity as an additional parameter could assist in the prediction of sumoylation sites. Currently, SUMOhydro is freely accessible at http://protein.cau.edu.cn/others/SUMOhydro/.

## Introduction

Sumoylation represents an important class of protein post-translational modifications (PTMs) in which a small ubiquitin-like modifier (SUMO) protein is covalently attached to a protein. By adding a SUMO protein to a substrate in a sequence-specific manner, protein sumoylation has the capacity to regulate multiple biochemical properties of the protein target, such as the stability, activity, intracellular localization and protein interactions. As such, sumoylation can play a critical functional role in various biological processes, including gene transcription and signal transduction [1], [2], []. Because most SUMO substrates are localized in the nucleus, protein sumoylation might have significant effects on nuclear functions [4], and sumoylation has been shown to be correlated with DNA damage recovery, gene expression and chromosomal integrity [5], [6]. In addition, the functional importance of protein sumoylation is reflected in a variety of human diseases, including Alzheimer's disease (AD), Parkinson's disease (PD) [7], viral infections [8] and cancers [9], [10].

SUMO proteins are widely expressed by all eukaryotes. In mammals, there are at least three SUMO proteins, SUMO1, SUMO2 and SUMO3, among which SUMO2 and SUMO3 are twin proteins [1]. In addition, SUMO4 has been identified but is expressed only in the kidneys and spleen [11]. Less advanced eukaryotes, such as yeast, worms and flies, express only a single SUMO gene. In plants, there are at least eight SUMO genes, and the reversible conjugation of SUMO to protein substrates has been demonstrated as a conserved regulatory process [12]. It has been well established that the consensus motif ψKxE (ψ represents a large hydrophobic amino acid, and x represents any amino acid) is essential for SUMO1 conjugation, and this motif has been intensively studied. In addition, two other extended consensus motifs have been recently identified. One motif is the PDSM (phosphorylation-dependent sumoylation motif), which is composed of a SUMO consensus site and an adjacent proline-directed phosphorylation site (ψKxExxSP) [13], and the other is known as an NDSM (negatively charged amino acid-dependent sumoylation motif) [14], which refers to the negatively charged amino acids that frequently appear within the 10 amino acids downstream of the core SUMO motif, ψKxE. Although these motifs might help define the majority of functional SUMO substrates, many types of sumoylation can not be classified according to these rules. For example, approximately 26% (95/370) of confirmed sumoylation events occur in non-consensus motifs. Thus, a better understanding of sequence-based prediction is necessary. Because sumoylation is reversible and unstable, there are significant limitations to experimental study designs, and labor-intensive methods are required; consequently, there has been an increasing interest in the computation-aided identification of sumoylation sites.

Currently, a number of elegant methods for predicting sumoylation sites are available. SUMOplot, which scores the sequence fragment xKxx in comparison to the consensus motif ψKxE, was the earliest publicly available web server for sumoylation site prediction. Subsequently, Xue et al. applied GPS and motifX in SUMOsp, which achieved a prediction sensitivity as high as 89.12% [15] and was updated to SUMOsp2.0 in 2009 [16]. An additional bioinformatics tool, SUMOpre, which uses a statistical method for sumoylation prediction, was also developed and yields an excellent prediction performance with a correlation coefficient of up to 0.636 [17]. Another classifier called seeSUMO has recently been developed for predicting sumoylation sites, which used the domain-specific knowledge in terms of relevant biological features for input vector encoding [18]. However, the prediction performances achieved by these existing methods require improvement mainly because the database employed in their tool development is limited and does not represent the full characteristics of SUMO substrates. For example, there are cases in which non-sumoylated proteins have always been falsely predicted to be sumoylated simply because they contain the consensus motif ψKxE. In other cases, proteins that lack this consensus motif may actually be sumoylated and not identified. Therefore, there remains a significant need to develop better predictors of protein sumoylation sites.

The input feature vector (i.e., encoding scheme) is critical in the development of predictors based on machine learning algorithms. An appropriate feature construction or encoding scheme is capable of reflecting the biological characteristics of sequence fragments, and common position-specific features, such as binary encoding, have been widely used as input features. Certain physicochemical properties of amino acids, such as hydrophobicity and solvent accessibility, have also been used as input features. An additional potentially useful encoding method is evolutionary information in the form of multiple sequence alignment profiles generated by the PSI-BLAST program [19]. The composition of k-spaced amino acid pairs (CKSAAP) has also been successfully employed to predict protein flexible/rigid regions [20], protein crystallization [21], protein structural classes [22] and mucin-type O-glycosylation sites [23], for example. These analyses are helpful in guiding the selection of novel encoding schemes for sumoylation sites prediction.

In the present study, SUMOhydro was developed to improve the prediction performance of sumoylation sites by seeking a new encoding scheme. After assessing various encoding schemes, we found that prediction performance could be improved by combining amino acid hydrophobicity with the binary encoding scheme. It has been proven that hydrophobicity plays a critical role in sumoylation site recognition. With the assistance of support vector machine (SVM), a widely used machine learning method, the leave-one-out cross-validation tests displayed an excellent performance with a Matthews' correlation coefficient (MCC), specificity, sensitivity and accuracy equal to 0.690, 98.6%, 71.1% and 97.5%, respectively. Finally, the SUMOhydro server was developed to define sumoylation sites in query proteins and is available online. Here, we present details on the construction of SUMOhydro, its overall performance and in-depth benchmark experiments against three of the current predictors.

## Results

### Prediction Performance

**Figure 1:**
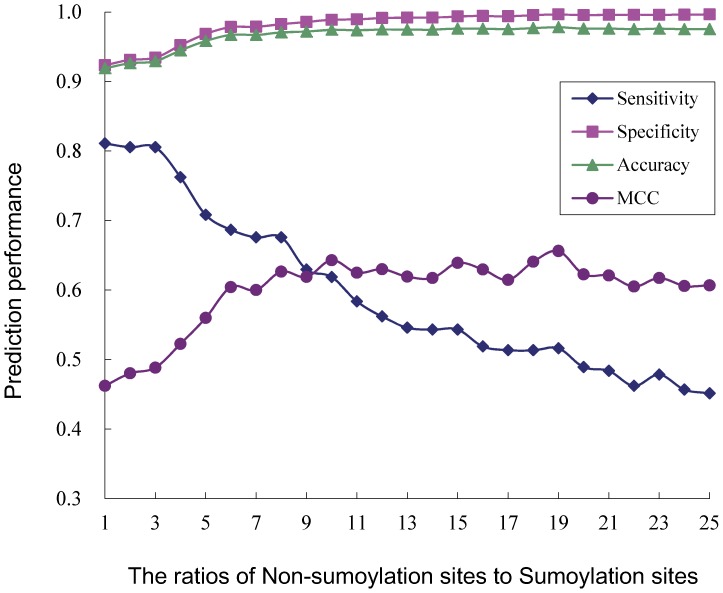
Prediction performance for different ratios of positive to negative sets based on binary encoding. The performance of the binary encoding scheme was assessed using a 10-fold cross-validation strategy.

The SUMOhydro predictor, which is based on a new, stringent sumoylation dataset, was constructed by employing the SVM algorithm. Because the ratio of sumoylation to non-sumoylation sites was significantly imbalanced (approximately 1:25) and because the SVM method, compared to other statistical methods, is highly sensitive to the ratio of positive to negative samples in the training dataset, the algorithm was trained on datasets with a series of different ratios of sumoylation to non-sumoylation sites, from 1∶1 to 1∶25 and was tested on the entire dataset. Details regarding the compilation of the datasets, encoding schemes and SVM algorithm are outlined in the Methods section. Four measurements [accuracy (*Ac)*, sensitivity (*Sn*), specificity (*Sp*) and MCC] were jointly used to assess the performance of the proposed sumoylation site predictor (*cf.* Table 1). Based on the MCC value of binary encoding, we determined 1∶10 as the final ratio of sumoylation sites to non-sumoylation sites in the training dataset (Figure 1). In these models, the window size was set at 25 (i.e., 2n+1 = 25) because this region could cover the 10-amino acid NDSM region located downstream from the core SUMO motif, ψKxE. For hydrophobicity-related encoding, the window size was imbalanced from 1 amino acid upstream to 2 amino acids downstream and was focused on the hydrophobic region.

**Table 1:** Prediction performance of 10-fold cross-validation based on different encoding methods.

Site	Encoding scheme	*Sn* (%)	*Sp* (%)	*Ac* (%)	MCC
K	Binary	60.3±2.1	98.8±1.4	97.2±0.1	0.631±0.005
	CKSAAP	55.7±2.4	94.6±0.1	93.0±0.1	0.385±0.026
	PSSM	51.1±2.2	95.8±0.1	93.9±0.0	0.393±0.022
	KNN	56.0±1.2	98.6±0.0	96.8±0.0	0.584±0.006
	Six_letter	53.5±3.8	96.1±0.2	94.3±0.0	0.422±0.035
	Nine_letter	57.9±3.1	95.4±0.1	93.8±0.1	0.426±0.027
	**Hydrobinary**	**61.0±3.7**	**99.3±0.1**	**97.7±0.1**	**0.682±0.018**
	Z_scales	57.5±3.2	98.6±0.1	96.8±0.1	0.593±0.017

a The SVM-based prediction algorithm was used, and the parameters for each encoding scheme were primary optimized. The hydrobinary encoding scheme resulted in the highest level of accuracy, and the corresponding *Sn*, *Sp*, *Ac* and MCC values are represented in bold. ^b^ Each corresponding measurement is represented as the average value ±standard deviation.

**Figure 2:**
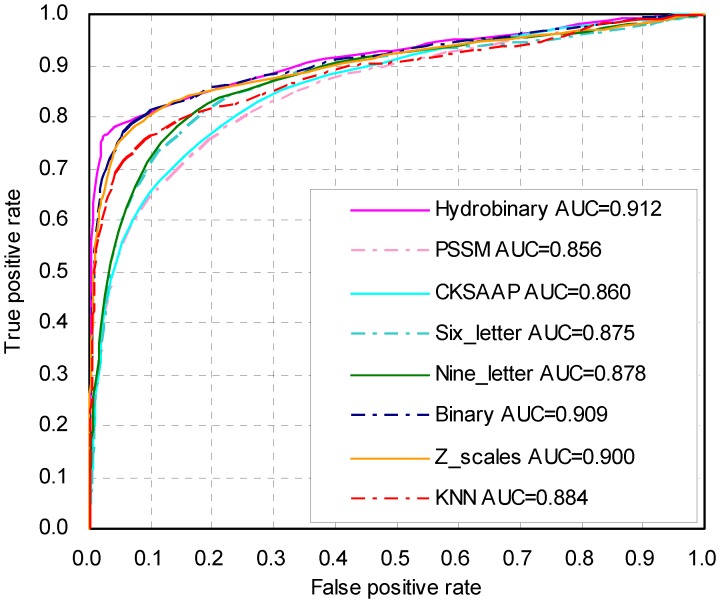
ROC curves of different encoding SVM models using a 10-fold cross-validation.

**Figure 3:**
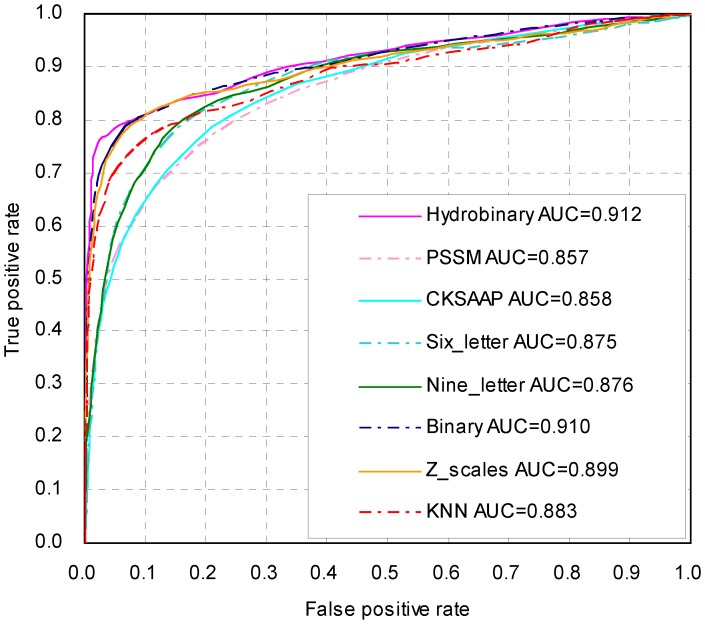
ROC curves of different encoding SVM models using a leave-one-out cross-validation.

Because there are always more non-sumoylation sites than sumoylation sites, we repeated the training/testing procedures 5 times by randomly changing the negative samples. When the number of positive and negative data points is different, the MCC should be more suitable for assessing the overall prediction accuracy. To test the stability of the hydrophobic encoding combined with the binary encoding, which was termed “hydrobinary encoding” in this study, we used two strategies on the same dataset: a 10-fold cross-validation and a leave-one-out cross-validation. The prediction performances are shown in Tables 1 and 2, with MCC values as high as 0.682 and 0.690. Because the dataset is highly imbalanced and the MCC can be affected by the tradeoff between sensitivity and specificity, the ROC curves for each strategy were plotted, and the corresponding AUC values were calculated (see Figures 2 and 3). Currently, the SUMOhydro web server is constructed based on the full dataset to facilitate research by the scientific community and is freely available at http://protein.cau.edu.cn/others/SUMOhydro/.

**Table 2:** Prediction performance of leave-one-out cross-validation based on different encoding methods.

Site	Encoding scheme	*Sn* (%)	*Sp* (%)	*Ac* (%)	MCC
K	Binary	59.3±0.6	99.0±0.0	97.3±0.1	0.640±0.005
	CKSAAP	57.0±1.9	93.7±0.1	92.1±0.1	0.367±0.016
	PSSM	55.3±1.4	94.8±0.1	93.1±0.1	0.388±0.021
	KNN	57.9±1.9	98.4±0.1	96.6±0.2	0.576±0.019
	Six_letter	58.3±4.8	95.2±0.5	93.7±0.1	0.423±0.020
	Nine_letter	57.4±3.0	95.2±0.2	93.6±0.3	0.415±0.021
	**Hydrobinary**	**71.1±2.9**	**98.6±0.1**	**97.5±0.2**	**0.690±0.017**
	Z_scales	60.1±4.6	98.4±0.1	96.8±0.4	0.599±0.037

a The SVM-based prediction algorithm was used, and the parameters of each encoding scheme were primary optimized. The hydrobinary encoding scheme resulted in the highest level of accuracy, and the corresponding *Sn*, *Sp*, *Ac* and MCC values are represented in bold. ^b^ Each corresponding measurement is represented as the average value ±standard deviation.

### Comparison of different Encoding Schemes

Eight encoding schemes have been utilized to represent the sumoylation site fragment, including traditional binary encoding, the composition of k-spaced amino acid pairs (CKSAAP) encoding, PSSM, KNN, six-letter, nine-letter, hydrobinary encoding and Z-scale encoding. To compare the performances of different encoding schemes, the predictors based on these different encoding schemes were trained and tested on our new datasets in parallel. As shown in Table 1, the commonly used binary encoding scheme performed much better compared to all other encoding schemes in a 10-fold cross-validation, with a MCC value of 0.631 (*Sn* = 60.3%, *Sp* = 98.8%, *Ac* = 97.2%). However, the binary encoding scheme was outperformed by a value of 0.051 (0.682 minus 0.631) when hydrobinary encoding was utilized. These results can be further illustrated in the receiver operating characteristic (ROC) curves and quantified using the corresponding areas under the ROC curves (AUC; Figure 2). Generally, the highest and leftmost ROC curve in the plot represents the best classification method. The ROC curve corresponding to hydrobinary encoding is the highest and leftmost curve, which reaches a maximum AUC value at 0.912 and represents a better result than that of binary encoding (Figure 2).

To further evaluate the performance of our method, a leave-one-out cross-validation was also performed, and the results are displayed in Table 2. The MCC of hydrobinary encoding persisted at a high level of 0.690, which was 0.050 greater than the MCC of binary encoding. ROC curves were plotted, and the corresponding AUC values were calculated (Figure 3). Because the results of the leave-one-out validation were almost identical to those of the 10-fold validation, the hydrobinary approach is a stable and robust encoding method for sumoylation prediction.

### Comparison of SUMOhydro with other Predictors

The proposed SUMOhydro method was benchmarked against three previously published sumoylation site predictors, SUMOpre [17], SUMOsp2.0 [16] and seeSUMO (http://bioinfo.ggc.org/seesumo/). SUMOpre uses a statistical method for sumoylation site prediction and was trained and tested on 268 sumoylation sites and 6361 non-sumoylation sites. When leave-one-out cross-validation was performed, the MCC, specificity, sensitivity and accuracy of SUMOpre were 0.636, 98.9%, 60.9% and 97.5%, respectively. Because the web server for SUMOpre was not available, SUMOpre could not be compared based on the independent test dataset. However, our method used a larger dataset and produced a better predictive performance. For example, the dataset presented here includes 358 sumoylation sites and 8071 non-sumoylation sites, and the leave-one-out cross-validation produced MCC, specificity, sensitivity and accuracy values of 0.690, 98.6%, 71.1% and 97.5%, respectively.

The local version of SUMOsp2.0, SUMOsp_2.0.4_windows_20090805.exe, was downloaded and used to predict 24 sumoylation sites in the independent test dataset. The only applicable thresholds used by SUMOsp2.0 were ‘low’, ‘medium’ and ‘high’, and three thresholds used in seeSUMO were -0.2 (low), 0 (medium) and 0.2 (high). To obtain analogous results, we set the corresponding thresholds of SUMOhydro to -0.2 (low), 0 (medium) and 0.2 (high). As shown in Table 3, although the sensitivity of SUMOsp2.0 and seeSUMO-RF achieved the highest performance at 75.0%, the specificity, accuracy and MCC of SUMOhydro reached higher performance with 91.4%, 90.5% and 0.405 in the low threshold predictions. For the medium threshold, the overall accuracy of seeSUMO is higher than SUMOsp2.0 and SUMOhydro; however, the MCC value of SUMOhydro is considerably higher than SUMOsp2.0 and seeSUMO. When the high threshold was used, the MCC value of SUMOhydro was 0.051 lower than that of SUMOsp2.0 with the same sensitivity at 58.3%. Because the sensitivity of SUMOhydro using the high threshold was relatively low, the optimal thresholds for practical applications would be low and medium. Based on these benchmarking results, we propose that SUMOhydro is a novel and useful tool for predicting sumoylation sites.

**Table 3:** Comparison of SUMOhydro with other predictors.

Threshold	Method	*Sn* (%)	*Sp* (%)	*Ac* (%)	MCC
Low	SUMOsp2.0	**75.0**	83.1	82.8	0.304
	seeSUMO-SVM	66.7	91.0	89.9	0.373
	seeSUMO-RF	**75.0**	82.8	82.4	0.300
	SUMOhydro	70.8	**91.4**	**90.5**	**0.405**
Medium	SUMOsp2.0	62.5	92.6	91.2	0.381
	seeSUMO-SVM	54.2	**95.1**	**93.3**	0.397
	seeSUMO-RF	**70.8**	88.4	87.6	0.351
	SUMOhydro	66.7	93.5	92.3	**0.432**
High	SUMOsp2.0	58.3	96.3	94.6	**0.470**
	seeSUMO-SVM	37.5	**97.8**	**95.1**	0.386
	seeSUMO-RF	**66.8**	90.4	89.3	0.362
	SUMOhydro	58.3	94.9	93.3	0.419

a SUMOhydro, seeSUMO and SUMOsp2.0 were tested using an entirely independent dataset. ^b^ The highest values for each threshold are indicated in bold.

## Discussion

A competitive sumoylation site predictor termed SUMOhydro was developed in the present study. We included amino acid hydrophobicity in a binary encoding scheme, and this hydrobinary encoding was proven suitable for the prediction of sumoylation sites, which gives SUMOhydro a better level of performance and favorable results relative to previously described predictors. Not only does its ability to clearly characterize amino acids in different positions surrounding a potential sumoylation site, it also pays attention on the biochemical property at different positions. It has been well known that more than two-thirds of the known sumoylation substrates have the consensus motif ψKxE, suggesting that sumoylation targets the substrate proteins at a specific position in most cases. Hence, we choose the position-specific binary encoding as one part of our hydrobinary encoding approach. On the other hand, the hydrophobicity has been proven to play a critical role in sumoylation site recognition. Therefore, the hydrobinary encoding is particularly suitable for the prediction of sumoylation. Although the overall function of this new tool remains unsatisfactory, we expect that the hydrobinary encoding approach reported here will be useful for the further development of more successful sumoylation prediction systems by adopting additional state-of-the-art machine learning methods or by combining this technique with other encoding schemes. The SUMOhydro web server has been constructed to facilitate its use by the biological community, and it is freely accessible at (http://protein.cau.edu.cn/others/SUMOhydro/). In conclusion, this tool has possible applications to proteome-wide sumoylation site prediction.

## Methods

### Datasets

The experimentally validated sumoylation sites were extracted from two sources. The first source was SUMOsp2.0, which contains 332 non-redundant sumoylation sites in 197 proteins compiled from research articles published prior to October 18, 2007. The second source was the PubMed database, which was searched using the keywords “SUMO” and “sumoylation” to obtain data from October 18, 2007, to January 16, 2010. This search found 38 experimentally defined sumoylation sites in 27 proteins from 362 research articles. These primary sequences were also extracted from the UniProt database (http://www.uniprot.org/) (see Supporting Information, Document S1). In total, 221 proteins, covering 370 sumoylation sites, were compiled into the initial positive dataset. Similar to other PTM sites predictors, the input for a sumoylation site predictor is also generally represented by a 2n+1 residue-long sequence with a K in the central position (i.e., the window size is equal to 2n+1). Each site within the datasets is represented by a sequence fragment of 25 amino acids where K is in the central position. For the sites located in the N- or C-terminus, the number of upstream or downstream residues may be less than 12. To ensure a sequence fragment with a unified length, a non-existent amino acid O was assigned to fill in the corresponding positions. Thus, in the present study, 21 amino acids were considered to reflect the sequence context of a sumoylation site, which were ordered alphabetically as ACDEFGHIKLMNPQRSTVWYO. All K residues in these 221 protein sequences with no annotation related to a sumoylation site were selected as negative sites. A total of 9195 non-sumoylation residues were initially selected. We further filtered the initial dataset using a threshold of 40% sequence identity to avoid an overestimation of the performance that would be caused by sequence redundancy. This procedure ensured that any given fragment pairs in all of the remaining positive and negative samples shared a sequence identity of less than 40%. Finally, we obtained a filtered sumoylation site dataset containing 358 positive (Positive_K) and 8071 negative samples (Negative_K), which were utilized to train and test SUMOhydro (see Supporting Information, Documents S2 and S3).

To independently compare the prediction performance of SUMOhydro with previous predictors, we used a test dataset consisting of an additional 24 sumoylation sites (Positive_test.txt) and 510 non-sumoylation sites (Negative_test.txt) in 17 proteins that were reported from June 1, 2010 to January 1, 2012 (see Supporting Information, Documents S4 and S5). This dataset excluded all the instances used by seeSUMO, including the sumoylation sites from research articles published before June 1, 2010.

### Feature Construction

#### Binary encoding

In the binary encoding scheme, each amino acid is represented by a 21-dimensional binary vector, e.g., A (100000000000000000000), C (010000000000000000000), …, O (000000000000000000001), etc. For a query sumoylation site represented by a fragment of 2*n*+1 residue, the central residue is always K, which does not need to be considered. Therefore, the total dimension of the proposed binary feature vector is 21×2*n*.

#### CKSAAP encoding

In this study, a sumoylation site is represented by a sequence fragment of 25 amino acids. CKSAAP encoding reflects the composition of *k*-spaced amino acid pairs (*i.e.*, pairs that are separated by *k* other amino acids) within this sequence fragment. A feature vector is then used to represent the composition of these pairs, which can be described as follows:
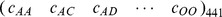
(1)


The value of each feature denotes the composition of the corresponding amino acid pair in the fragment. For example, if an AD pair appears m times in a fragment, the composition of the AD pair in the vector (*i.e.*,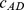) is equal to m. The amino acid pairs for *k* = 0, 1, …, *k_max_* are jointly considered in this study. Therefore, the total dimension of the proposed feature vector is 441× (*k_max_*+1). In our study, we define the *k_max_* as equal to 4 when considering the dimension and overall performance.

#### PSSM encoding

The PSSM-encoding method has consistently been used to predict biological problems because of its ability to reflect the evolutionary information of a sequence fragment, such as the prediction of RNA-binding sites [24], [25] and subcellular localizations of Gram-negative bacterial proteins [26]. The input for each sequence fragment consisted of a corresponding row in the position-specific scoring matrix (PSSM) generated from three cycles of PSI-BLAST [19] searches against the Swiss-Prot non-redundant database using an E-value of 0.001.

#### KNN encoding

KNN encoding scheme is based on the concept of the nearest neighbor algorithm, which is a method for classifying a new object according to the *k* closest samples in the feature space [27]. The detailed procedures of this encoding method are described as follows. For two sequence fragments S_1_ {aa_1_, aa_2_, …, aa_n-1_, aa_n_} and S_2_ {aa_1_, aa_2_, …, aa_n-1_, aa_n_}, their distance (S_1_, S_2_) can be defined as:.
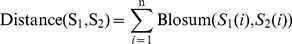
(2)where n is the length of the sequence fragment, and the amino acid similarity matrix is derived from normalized BLOSUM62 matrix [28]. The average distances from the new sequence *s* to the *k* nearest neighbors in the positive and negative sets were then calculated and denoted as *D_p_* and *D_n_*, respectively. The KNN score is defined as the ratio of *D_p_* to *D_n_*. Finally, we set a different *k* value to obtain a series of KNN features. In this study, *k* was chosen to be (1, 3, 5, 7, 9, 15).

#### Six_letter encoding

Six_letter encoding is a simple binary encoding method that uses a reduced alphabet. The 20 amino acids were divided into five groups based on their physical characteristics, which were aliphatic (alanine, valine, leucine, isoleucine), charged (aspartic acid, glutamic acid, arginine, lysine), polar (serine, threonine, asparagine, glutamine), cyclic (phenylalanine, histidine, tyrosine, tryptophan) and other (glycine, proline, methionine, cysteine). Because we defined a nonexistent amino acid “O” to represent an empty position, we obtained a total of six groups.

#### Nine_letter encoding

This encoding scheme is similar to six_letter encoding; however, the group “other” was expanded into individual amino acids. Thus, the nine_letter encoding scheme included the groups: aliphatic, charged, polar, cyclic, glycine, proline, methionine, cysteine and the nonexistent amino acid “O”.

#### Hydrobinary encoding

A new feature construction, termed “hydrobinary encoding”, was employed. The basic premise of this encoding scheme is to combine hydrophobicity and binary encoding. In this encoding scheme, each amino acid is represented by a corresponding value, which is based on a hydrophobicity scale matrix (*cf.* Table 4). The window size used for hydrophobicity encoding is 1 amino acid upstream to 2 amino acids downstream, which covers the entire hydrophobic region. In addition, each amino acid is also represented by binary encoding. The window size for the binary encoding used here is the same as for binary encoding used independently. Therefore, the total dimension of this proposed method is 21×2n+1×4.

**Table 4:** Hydrophobicity scales for the 20 amino acids.

Amino Acid	Feature Value	Amino Acid	Feature Value
A	1.81	M	2.35
C	1.28	N	-6.64
D	-8.72	P	4.04
E	-6.81	Q	-5.54
F	2.98	R	-14.92
G	0.94	S	-3.40
H	-4.66	T	-2.57
I	4.92	V	4.04
K	-5.55	W	2.33
L	4.92	Y	-0.14

a Cited from [35] **.**

#### Z_scales encoding

In this encoding scheme, each amino acid is characterized by five physicochemical descriptor variables (*cf.* Table 5), which were developed by Sandberg et al. in 1998 [29].

**Table 5:** Z_scale for the 20 amino acids.

Amino Acid	z_1_	z_2_	z_3_	z_4_	z_5_	Amino Acid	z_1_	z_2_	z_3_	z_4_	z_5_
A	0.24	-2.32	0.60	-0.14	1.30	M	-2.85	-0.22	0.47	1.94	-0.98
C	0.84	-1.67	3.71	0.18	-2.65	N	3.05	1.62	1.04	-1.15	1.61
D	3.98	0.93	1.93	-2.46	0.75	P	-1.66	0.27	1.84	0.70	2.00
E	3.11	0.26	-0.11	-3.04	-0.25	Q	1.75	0.50	-1.44	-1.34	0.66
F	-4.22	1.94	1.06	0.54	-0.62	R	3.52	2.50	-3.50	1.99	-0.17
G	2.05	-4.06	0.36	-0.82	-0.38	S	2.39	-1.07	1.15	-1.39	0.67
H	2.47	1.95	0.26	3.90	0.09	T	0.75	-2.18	-1.12	-1.46	-0.40
I	-3.89	-1.73	-1.71	-0.84	0.26	V	-2.59	-2.64	-1.54	-0.85	-0.02
K	2.29	0.89	-2.49	1.49	0.31	W	-4.36	3.94	0.59	3.44	-1.59
L	-4.28	-1.30	-1.49	-0.72	0.84	Y	-2.54	2.44	0.43	0.04	-1.47

### Support Vector Machine (SVM)

An SVM is a machine learning algorithm that has been widely employed for different biological problem predictions, such as protein fold recognition [30], protein isomerization classification [31] and the prediction of membrane protein types [32]. Generally, SVM constructs a hyperplane in a high-dimension space, which separates two different groups of feature vectors in the training set using a maximum margin. The implementation of the SVM algorithm used here was SVM-Light (http://svmlight.joachims.org/). In the current study, two parameters (i.e., the regularization parameter *C* and the width parameter *γ*) in a radial basis function (RBF), which is one of the kernel functions in SVM, were determined in advance to optimize the SVM training.

### Performance Assessment

Four measurements, *i.e.*, *Ac*, *Sn*, *Sp* and MCC, which are commonly used in other studies, were applied to evaluate the prediction performance. The definitions are as follows:
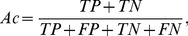
(3)

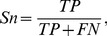
(4)

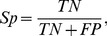
(5)

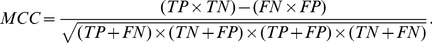
(6)where *TP*, *FP, FN* and *TN* denote true positives, false positives, false negatives and true negatives, respectively. Among these values, the MCC value is the most important measurement when considering the highly imbalanced training dataset used here. The MCC value ranges from -1 to 1, and a higher value indicates a better prediction performance.

The prediction accuracy was also measured using an ROC analysis [33], [34], which plots the true positive rate (*i.e.*, *Sn*) as a function of the false positive rate (*i.e.*, 1-*Sp*) for all possible thresholds. The area under the ROC curve (AUC) was also calculated to quantify the prediction performance of the proposed method. Generally, a prediction method is considered to improve as the AUC value approaches 1.

## Supporting Information

Document S1 Document S1 contains the primary sequences of the sumoylated proteins extracted from the UniProt database.
(FA) Click here for additional data file.

Document S2 Document S2 contains the sumoylation sites (Positive_K) used for training and testing the proposed SUMOhydro predictor.
(TXT)

Document S3 Document S3 contains the non-sumoylation sites (Negative_K) used for training and testing the proposed SUMOhydro predictor.
(TXT)

Document S4 Document S4 contains the sumoylation sites (Positive_test.txt) that were used in the independent test dataset.
(TXT)

Document S5 Document S5 contains the non-sumoylation sites (Negative_test.txt) that were used in the independent test dataset.
(TXT)
